# Renal Thrombotic Microangiopathy: A Complication of Paediatric Systemic Lupus Erythematosus That Requires Attention

**DOI:** 10.7759/cureus.48382

**Published:** 2023-11-06

**Authors:** Pranjal Kalita, Vandana Raphael, Biswajit Dey, Himesh Barman, Nirvana Thangjam, Donboklang Lynser, Kapil Dev Rabha, Amrita Das

**Affiliations:** 1 Pathology, North Eastern Indira Gandhi Regional Institute of Health and Medical Sciences, Shillong, IND; 2 Pediatrics, North Eastern Indira Gandhi Regional Institute of Health and Medical Sciences, Shillong, IND; 3 Radiology, North Eastern Indira Gandhi Regional Institute of Health and Medical Sciences, Shillong, IND; 4 Pediatrics and Neonatology, All India Institute of Medical Sciences, Bhopal, IND; 5 Radiology, Tezpur Medical College, Tezpur, IND

**Keywords:** plasmic score, renal vascular lesions, class iv lupus nephritis, paediatric systemic lupus erythematosus, renal thrombotic microangiopathy

## Abstract

Systemic lupus erythematosus (SLE) is a multi-system disorder with a variety of clinical presentations. A wide range of renal vascular lesions (VL) is described predominantly in adult patients. The exact prevalence of renal VL in the pediatric SLE (pSLE) population is yet to be determined. A 10-year-old female patient with lupus nephritis (LN) presented with deteriorating kidney function. An exhaustive array of clinical-biochemical and pathological evaluations resulted in a diagnosis of class IV LN with thrombotic microangiopathy (TMA) associated with malignant hypertension and hypocomplementemia. Renal VL is overlooked or underreported in SLE patients, as it is neither accorded much importance in the International Society of Nephrology/Renal Pathology Society (ISN/RPS) classification nor in the activity and chronicity scoring. The TMA lesions in LN patients can be managed following the recently devised PLASMIC score; hence, reporting such VL has therapeutic implications.

## Introduction

Pediatric systemic lupus erythematosus (pSLE) is an autoimmune chronic inflammatory disorder with multi-system involvement with onset before 18 years of age, accounting for approximately 15% of patients diagnosed with SLE amongst all groups [1]. Approximately 50% to 75% of patients with pSLE have renal involvement within two years of diagnosis [2]. All compartments of the kidney can be involved in lupus nephritis (LN). However, renal vascular lesions (VL) are generally overlooked, although a variety of renal vessel lesions have been described in the medical literature in SLE patients [3]. The exact prevalence of vasculitic lesions in pSLE is undocumented in spite of its aggressive presentation and its presence bearing a poor prognostic implication [4].

We report a case of thrombotic microangiopathic (TMA) lesion in a 10-year-old female suffering from LN who presented to a tertiary care center with a variety of clinical symptoms. The patients fulfilled the 2019 American College of Rheumatology/European League Against Rheumatism (ACR/EULAR) criteria for the classification of SLE. The authors of the case report want to create awareness about the fact that TMA or any VL in pSLE patients are often overlooked and underreported because the presence of such lesions does not affect the International Society of Nephrology and Renal Pathology Society (ISN/RPS) categorization of LN nor bear much impact on the activity and chronicity indices. Treatment received by the patient with concomitant LN and TMA lesions may be impacted and ultimately modified as dictated by the PLASMIC score developed by Bendapudi et al.; hence, reporting such VL is of utmost importance [5].

## Case presentation

A 10-year-old female was referred to the Department of Paediatrics in a tertiary care institute with complaints of generalized weakness for one month, pain in the abdomen, reddish-colored urine, swelling of bilateral lower limbs for 11 days, and fever for eight days. On examination, the patient was pale, febrile, tachypneic, anicteric, and had stage 2 hypertension (186/120 mmHg). The pulse rate was 110 beats per minute, normal in character and volume; the rhythm was regularly regular with no radio-radial or radio-femoral delay; and all the peripheral pulses were palpable. The patient also had features of hypertensive encephalopathy in the form of headache, blurring of vision, vomiting, and episodes of generalized tonic-clonic seizures and had a Glasgow coma scale (GCS) score of 10/15. No signs of trauma, bruises, or bleeding were noted. All developmental milestones were achieved at an appropriate age. There was a history of receiving three units of packed red blood cells before being referred to our hospital. The patient was admitted to the pediatric intensive care unit (PICU) with multiple differential diagnoses ranging from secondary glomerulonephritis to atypical hemolytic uremic syndrome that were considered, and relevant laboratory investigations were conducted (Table 1).

**Table 1:** Relevant laboratory findings in the patient

Laboratory Parameters	Patient Values	Reference Range with Units
Complete blood count and peripheral blood smear findings		
Hemoglobin (gm%)	10.3	12-15 gm%
Total leucocyte count (/L)	5000	4500-13500/L
Differential leucocyte count (%)	55 (neutrophils), 40 (lymphocytes), 03 (monocytes), 02 (eosinophils)	Neutrophils: 23%-53%, Lymphocytes: 23%-53%, Monocytes: 2%-11%, Eosinophils: 1%-4%
Platelet count (/mcL)	320,000	150,000-450,000/mcL
Reticulocyte count (%)	1.07	0.5%-1.5%
Peripheral blood smear finding	Normocytic normochromic anaemia. No signs of hemolysis noted.	Within normal limits for age
Biochemical findings		
Serum Urea (mg/dL)	170	17-45 mg/dL
Serum Creatinine (mg/dL)	3.9	0.6-1.4 mg/dL
Serum Sodium (mmol/L)	136	135-145 mmol/L
Serum Chlorine (mmol/L)	105	98-108 mmol/L
Serum Potassium (mmol/L)	3.5	3.5-5.5 mmol/L
Estimated glomerular filtration rate (eGFR) by Schwartz equation (mL/min/1.73m^2^)	17	>90 mL/min/1.73m^2^
C-reactive protein (CRP) (mg/dL)	1.14	0.00-0.800 mg/dL
Erythrocyte sedimentation rate (ESR) (mm/ 1hour)	37	0-20mm/1hr
Urine analysis		
Protein	2+	Nil
Red blood cells	99/hpf	Nil

**Figure 1:**
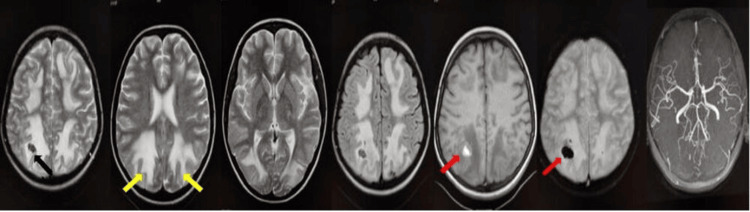
MRI studies of the patient Black arrow: Posterior reversible encephalopathy syndrome with subacute intracerebral hemorrhage; Yellow arrow: Symmetrical periventricular, largely posteriorly located high T2/FLAIR signal is noted; Red arrows: T1 hyperintense focus showing blooming on GRE sequence in right parietal lobe suggestive of hemorrhage FLAIR: Fluid-attenuated inversion recovery, GRE: Gradient recalled echo

The child tested positive for antinuclear antibody (ANA) and double-stranded DNA (dsDNA) and was negative for antineutrophil cytoplasmic antibody (ANCA) antibodies. Serological investigations for malaria, typhoid, hepatitis B surface antigen (HBsAg), anti-hepatitis C virus (anti-HCV), and HIV were negative. Direct Coombs test results were negative, whereas ADAMTS13 levels were within the normal range. Magnetic resonance imaging studies showed symmetrical bilateral parieto-occipital white matter hyperintensity. A radiological diagnosis of posterior reversible encephalopathy syndrome (PRES) was favored (Figure 1).

Initial laboratory evaluation revealed near-normal hemoglobin (post-transfusion) and a normal total count, platelet count, and reticulocyte count. The cause of anemia requiring blood transfusions was obscure. A positive ANA and dsDNA, along with the presenting features, established a diagnosis of SLE (a score of 31 as per the ACR/EULAR 2019 criteria). The child was managed with intravenous labetalol targeting blood pressure as per hypertensive emergency protocol. The episodes of seizures were managed by sodium valproate. She was simultaneously pulsed with methylprednisolone owing to acute kidney injury and a stormy acute phase. The sensorium improved upon control of blood pressure, and renal function improved over days. Once the child stabilized, a renal biopsy was planned.

**Figure 2:**
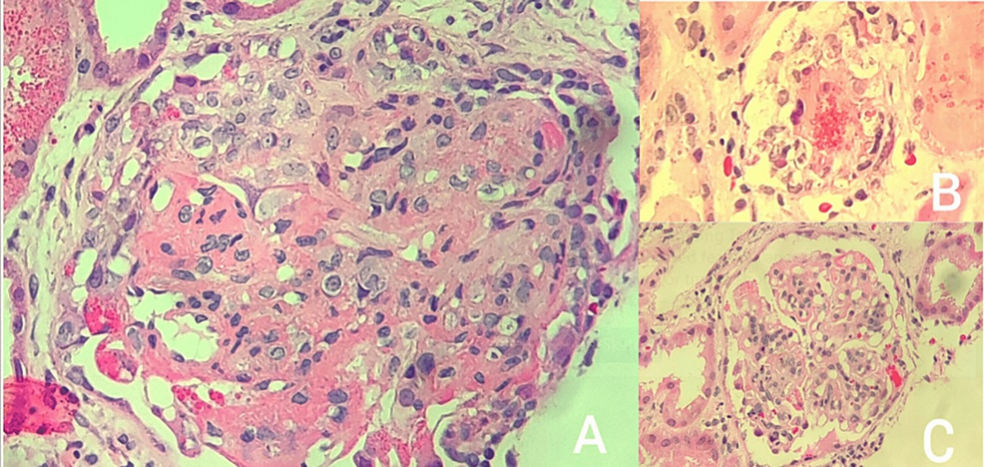
Light microscopy evaluation A: Glomerular capillary congestion, endothelial swelling and necrosis and glomerular capillary thrombosis with entrapment of fragmented erythrocytes (H&E, 200x); B: Vessel wall with luminal occlusion, fibrin deposition and fragmented RBCs (H&E, 200x); C: Diffuse LN lesion in glomerulus (H&E, 200x) H&E: Hematoxylin and eosin, LN: Lupus nephritis

**Figure 3:**
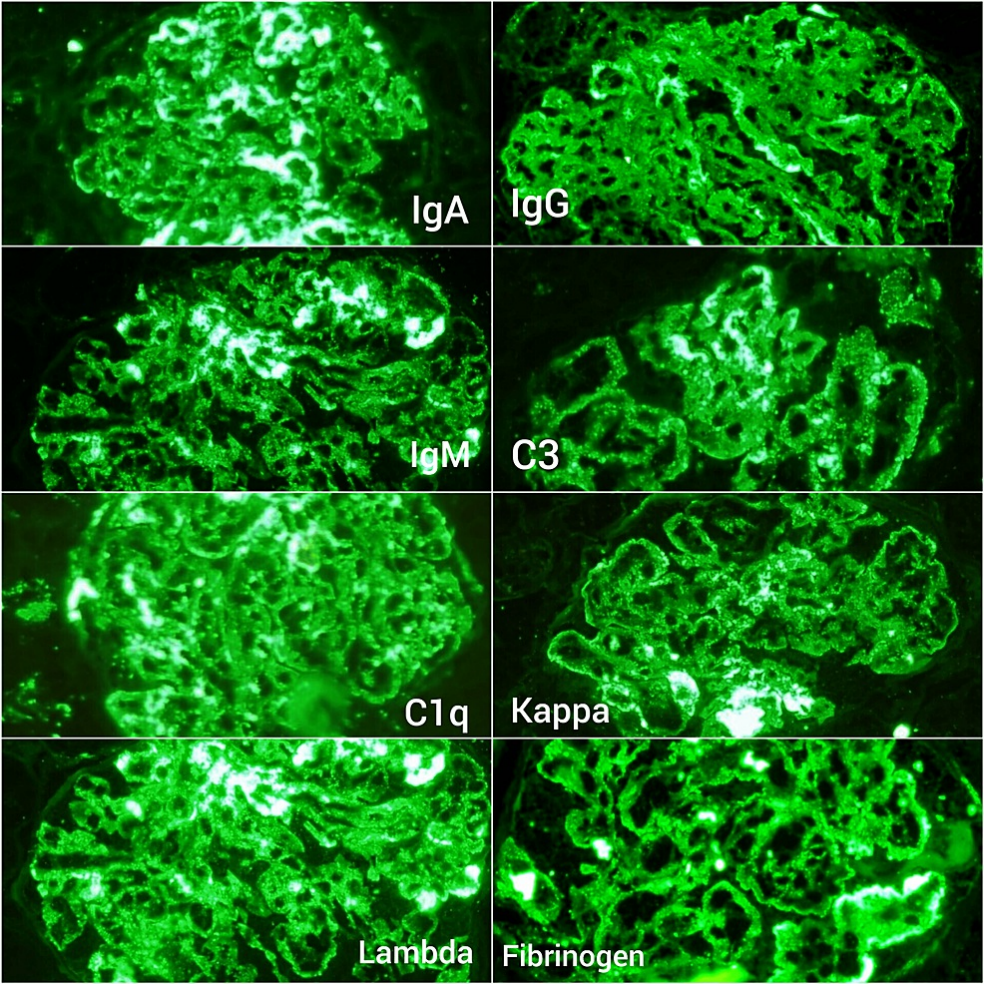
Full house pattern of positivity characteristic of LN along with fibrin deposition demonstrated by immunofluorescence suggestive of intraglomerular thrombi (IF, 200x) LN: Lupus nephritis, IF: Immunofluorescence

Light microscopy evaluation of the renal biopsy revealed 11 glomeruli, with eight glomeruli showing mesangial matrix expansion and hypercellularity along with endocapillary hypercellularity. Seven glomeruli showed wire-loop lesions. One glomerulus showed global sclerosis, and one glomerulus showed a fibrous crescent. Glomerulus also showed capillary congestion, endothelial swelling, necrosis, and glomerular thrombosis with the entrapment of fragmented erythrocytes. Tubules showed evidence of mild atrophy and the presence of an RBC cast. The interstitium showed infiltration by chronic inflammatory cells. Interstitial fibrosis and tubular atrophy (IFTA) was approximately 35%. Occasional blood vessels showed vessel walls with luminal occlusion, fibrin deposition, and fragmented RBCs. Immunofluorescence showed granular immunostain deposits of IgA, IgG, IgM, C3, C1q kappa, and lambda. Fibrinogen immunostaining showed 3+ positivity in the glomerular thrombi, confirming the presence of fibrin deposits. However, the vessels did not show any immune deposits. Correlating the histomorphology and immunofluorescence findings, a diagnosis of class IV LN with thrombotic microangiopathy was favored following the ISN/RPS classification. The activity and chronicity indices scores were 14/24 and 5/12, respectively (Figures 2-3).

Following the five doses of methylprednisolone pulse therapy and considering the diagnosis to be class IV LN, the patient was started on oral prednisolone, hydroxychloroquine, and mycophenolate mofetil (MMF). The serum urea and creatinine levels of the patient at discharge were 40 mg/dL and 0.8 mg/dL, respectively. The patient was followed up with a visit to the pediatrics outpatient department after one month, where the child was found to be doing well with normal renal function. However, she had not attained renal remission yet. So, she continues to be followed up.

## Discussion

Renal VL in patients with adult SLE is not an uncommon finding and is often associated with poor prognostic implications. The incidence of VL ranges from 10% to 40%, according to various studies [3]. In a review article published in 1994 by Appel et al., VL was categorized into the following categories: (1) uncomplicated vascular immune deposits; (2) lupus vasculopathy, also known as non-inflammatory necrotizing vasculopathy; (3) thrombotic microangiopathy; (4) necrotizing vasculitis; and (5) non-specific arteriosclerosis [6]. However, medical literature on the incidence and prevalence of VL in the pediatric population is extremely limited, and the exact data is yet to be documented. A total of 58 native pediatric renal biopsies were diagnosed as pediatric SLE in our institute (North Eastern Indira Gandhi Regional Institute of Health and Medical Sciences, Shillong, India) over 10 years (2012 to 2022). A total of 12 (20.6%) cases showed various renal vasculopathy lesions; however, a single case (8.3%) of renal TMA in pSLE was reported among these 12 cases, highlighting the rarity of renal thrombotic microangiopathic lesions in the pediatric study population. The adult population during the same study period, however, showed a slightly higher presentation of renal TMA, accounting for 13.8% of all renal vasculopathic lesions.

The pathogenesis of VL in SLE is yet to be completely understood, with both genetic, environmental, epigenetic, and hormonal influences playing their part. Endothelial activation secondary to immune dysregulation seems to be the initial event in renal VL in patients with SLE. The formation of autoantibodies after immune complex formation results in injury to the endothelial cell lining. Subsequent immune cell recruitment triggers an inflammatory response with an interplay of the complement cascade, adhesion molecule expression on the injured endothelium, various cytokines, and chemokines inducing various VL [7]. Various studies have evaluated the role of circulating endothelial cells and endothelial protein C receptors as predictors of VL, yet renal biopsy evaluation remains the gold standard of investigation for the detection of any renovascular lesions [3].

Wu et al. described the prevalence of VL in LN in the Chinese population aged 32.9±11.4 (mean ±SD) years. True vasculitic lesions accounted for 0.6%, whereas TMA accounted for 17.6%, compared to patients presenting with uncomplicated immune deposits in the vessels, constituting 74.2% of the study population, which highlights the rarity of true vasculitic lesions and thrombotic microangiopathic VL [8]. Song et al. described renal thrombotic microangiopathies in 36 cases out of 148 patients with LN, highlighting that TMA lesions are not entirely uncommon in LN patients and that they present with higher proteinuria, serum creatinine levels, higher activity, and chronicity indices [9].

The pathogenesis of TMA in LN is unclear. Both the classic and alternate complement pathways have been implicated in the injury and inflammatory reactions noted in SLE. Hypocomplement levels of C3 and C4 favor the involvement of an alternate pathway [10]. The TMA lesions in LN may be associated with a variety of diseases, namely anti-phospholipid syndrome (APS), scleroderma, calcineurin inhibitors (CI), and thrombotic thrombocytopenic purpura-hemolytic uremic syndrome (TTP-HUS). In our described case, various differential diagnoses were sequentially ruled out, aided by physical evaluation and laboratory investigations. Negativity to anti-phospholipid antibodies (APLA) ruled out TMA associated with APLA syndrome. The absence of any signs or symptoms of scleroderma ruled out the differential diagnosis of scleroderma. There was no history of calcineurin inhibitor intake. A differential diagnosis of TTP-HUS was also considered due to the presence of neurological symptoms along with acute kidney injury and anemia requiring transfusion. However, the absence of thrombocytopenia, findings of microangiopathic hemolytic anemia in peripheral blood examination, and normal ADAMTS13 level didn’t let us establish the diagnosis. Diagnoses of TMA associated with malignant hypertension with hypocomplementemia or direct small vessel endothelial injuries due to lupus activity were possibilities. However, there were no signs of immune-mediated vascular deposits. Therefore, we opine that hypertensive-related damage with consequent complement activation is primarily responsible for the TMA lesions in our case.

The case described had higher activity and chronicity indices and a stormy renal picture at presentation, a finding similar to studies done by Song et al. and Azarniouch et al. [9,11]. This highlights the fact that the overlooked vascular compartment in both ISN/RPS grading systems for categorization of LN into various classes and activity and chronicity indices needs further modifications to include VL. In today’s era of evidence-based targeted therapy, the management of patients with LN and accompanying TMA is based primarily on the PLASMIC score developed by Bendapudi et al. [5]. The authors of the present study acknowledge the utility of the PLASMIC score, and because ADAMTS13 levels were normal and negative for APLA, they are of the opinion that the best treatment modality for complement-mediated TMA and LN in our patient would be to use eculizumab, a monoclonal antibody. However, considering the resource constraints and unavailability of eculizumab, the patient could not be treated with the same

## Conclusions

The TMA lesions in pSLE patients are one of the various VL that can present with a grim renal presentation. Although described mostly in the adult population, its presence in pSLE patients is slowly getting recognized. The categorization of LN and the activity and chronicity indices that determine treatment and patient responses, along with disease progression, overlook the vascular compartment. Further modifications in these areas might have diagnostic, therapeutic, and prognostic implications. With the advent of a scoring system and an algorithm to guide management, it is of utmost importance to recognize such VL, and a combined clinical-biochemical and pathological approach is of utmost importance in such cases.
